# Granular cell tumour of the pancreas: a case report and systematic review

**DOI:** 10.1007/s00423-023-02761-3

**Published:** 2023-01-25

**Authors:** Kevin Tree, Krishna Kotecha, Shreya Mehta, Talia L. Fuchs, Christopher W. Toon, Anthony J. Gill, Jaswinder S. Samra, Anubhav Mittal

**Affiliations:** 1 Upper Gastrointestinal Surgical Unit, Royal North Shore Hospital, St Leonards, NSW Australia; 2 Present Address: Sydney Medical School Northern, University of Sydney, Sydney, NSW Australia; 3 Cancer Diagnosis and Pathology Group, Kolling Institute, Royal North Shore Hospital, St Leonards, NSW Australia; 4 University of Notre Dame Australia, Fremantle, Australia; 5 School of Medicine and Public Health, University of Newcastle, Callaghan, Australia

**Keywords:** Granular cell tumour, Pancreas, Systematic review, Diagnosis, Investigation

## Abstract

**Purpose:**

Granular cell tumours (GCTs) of the pancreas are mostly benign and exceptionally rare, with no unique identifying radiological features. Following a case discussion of a patient with GCT, a comprehensive review of available literature was conducted to identify the common diagnostic features associated with GCT.

**Methods:**

Following a case report identified in our institution, a systematic review was conducted by two authors in accordance with Preferred Reporting Items for Systematic review and Meta-Analysis protocols (PRISMA) guidelines. Databases MEDLINE, EMBASE, Scopus, World of Science, and grey literature were searched on August 2021. Inclusion criteria were histopathology diagnosed granular cell tumour of the pancreas.

**Results:**

A 37-year-old male presented with 1 month of abdominal pain and an MRI demonstrating a dilated main pancreatic duct, distal parenchymal atrophy, but no focal lesion. Repeat MRI at 6 months re-demonstrated similar findings and subsequent endoscopic ultrasound was suspicious for main duct IPMN. Following multidisciplinary team discussion, a spleen-preserving distal pancreatectomy was performed. Histopathology demonstrated granular cell tumour with cells diffusely positive for S100 and no malignant transformation.

11 case reports were identified in the literature with diagnosis confirmed on tissue histopathology based on positive immunohistochemical staining for S-100 protein. Eight patients presented with gastrointestinal symptoms with abdominal pain the main presenting complaint (50%). 10 patients underwent CT with portal venous contrast and all underwent endoscopic examination. Imaging findings were similar in five studies for EUS which demonstrated a hypoechoic lesion with homogenous appearance. On non-contrast CT GCT was iso-enhancing, and with portal venous contrast demonstrated hypo-enhancement that gradually enhanced on late phases. Pre-operative diagnosis of pancreatic carcinoma was described in six cases based on imaging and biopsy, resulting in progression to surgical resection. Nine patients were managed surgically and no complications identified on follow-up (6–52 months).

**Conclusion:**

The currently proposed management pathway includes EUS with biopsy and CT, and surgical resection recommended due to malignancy risk. Improved sample collection with EUS-FNA and microscopic assessment utilising S-100 immunohistochemistry may improve pre-operative diagnosis. Limitations include rare numbers in reported literature and short follow-up not allowing an assessment of GCT’s natural history and malignancy risk. Additional cases would expand the current dataset of GCTs of the pancreas, so that surgical resection may be avoided in the future.

## Introduction

Granular cell tumours (GCT) are rare tumours of Schwann cell origin and, whilst they are most commonly benign, malignancy has been reported in less than 2% of cases [1]. First reported by Alexis Abriskossof in 1926, GCTs have previously been referred to as Abrikossoff tumours, “granular cell myeloblastomas”, “granular cell neurofibromas”, and “granular cell schwannomas” [2]. GCT can occur anywhere in the body, including the chest, respiratory, and gastrointestinal tracts and central nervous system but most commonly occur in the tongue [3, 4]. There is recent evidence that the majority of GCT are driven by somatic mutations in the V-ATPase accessory genes ATP6AP1 and ATP6AP [5, 6]. GCT arising in the gastrointestinal tract is rare, accounting for only 5% of cases [2] and origin in the pancreas is particularly rare. Diagnosis of GCT is predominantly made on histopathological examination post resection; however, imaging investigations are often performed initially to distinguish from other benign or malignant lesions. No unique radiological characteristics exist for GCT due to heterogenous imaging findings, with distinguishing features dependent on anatomical location such as intramuscular or breast [7]. This study presents a case report of pancreatic GCT with a systematic review of the literature examining the characteristics, diagnosis, radiological findings, and management of pancreatic GCT.

## Case report

**Fig. 1:**
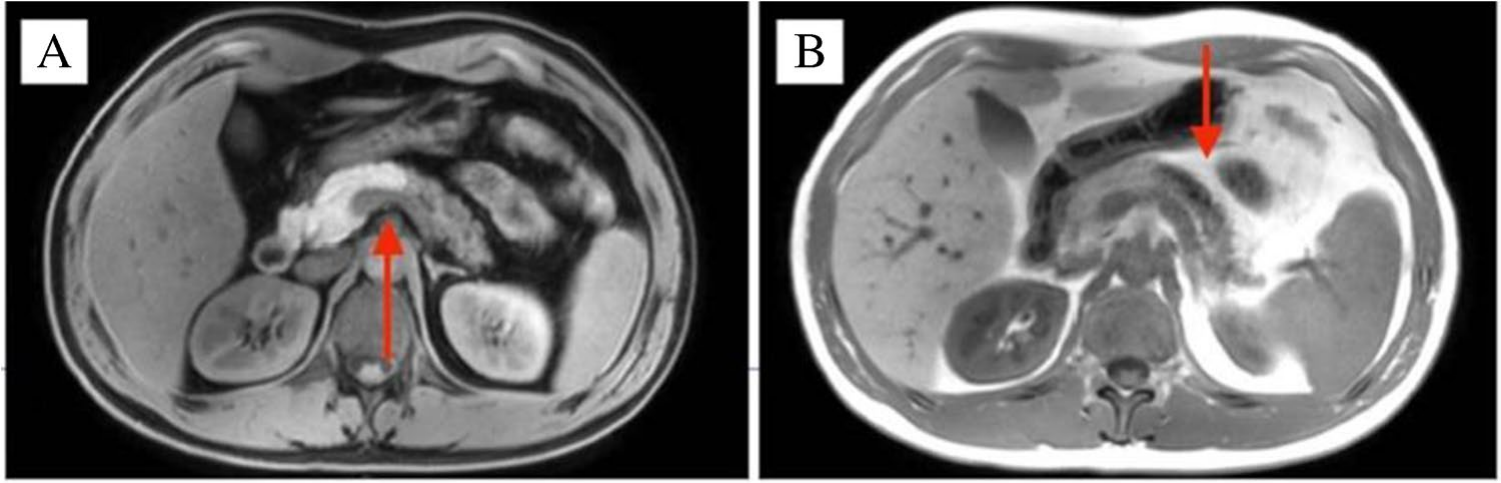
MRI scans demonstrating pancreatic main duct dilatation and adjacent pancreatic parenchymal atrophy and ductal dilatation: (**A**) axial T1-weighted image in the pre-contrast phase with hypoenhancement of the body and tail of pancreas and (**B**) T2-weighted in-phase scan with prominence of the dilated main pancreatic duct and mild increase in the enhancement of the surrounding parenchyma

**Fig. 2:**
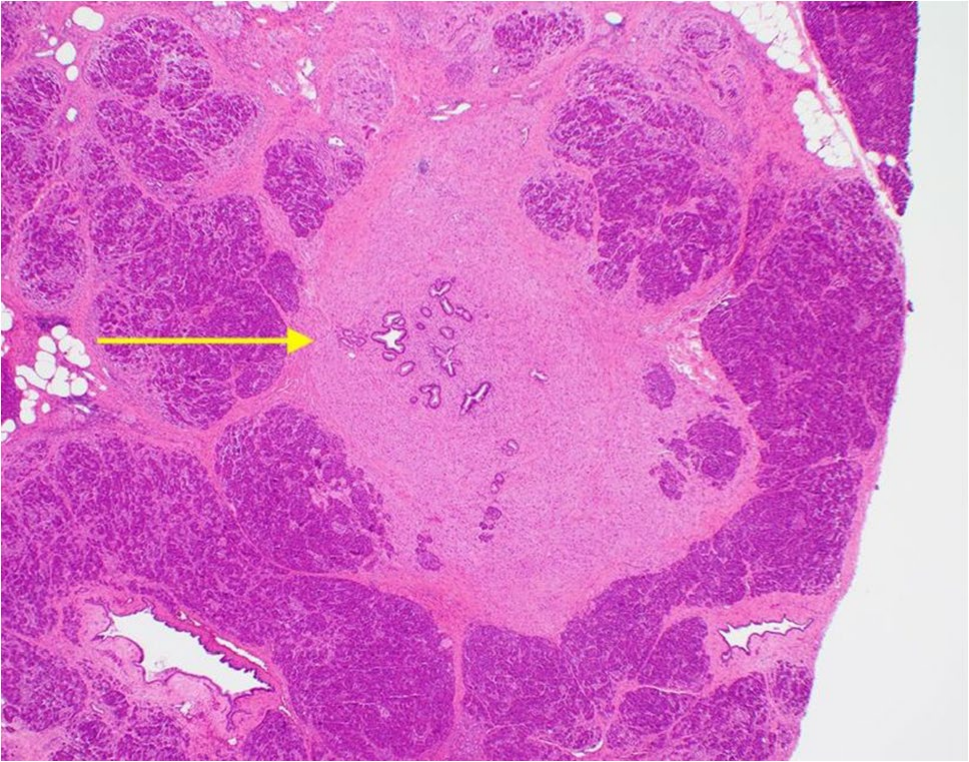
Section of pancreas showing small benign pancreatic ducts surrounded by granular cell tumour (H&E stain, 40 × magnification)

**Fig. 3:**
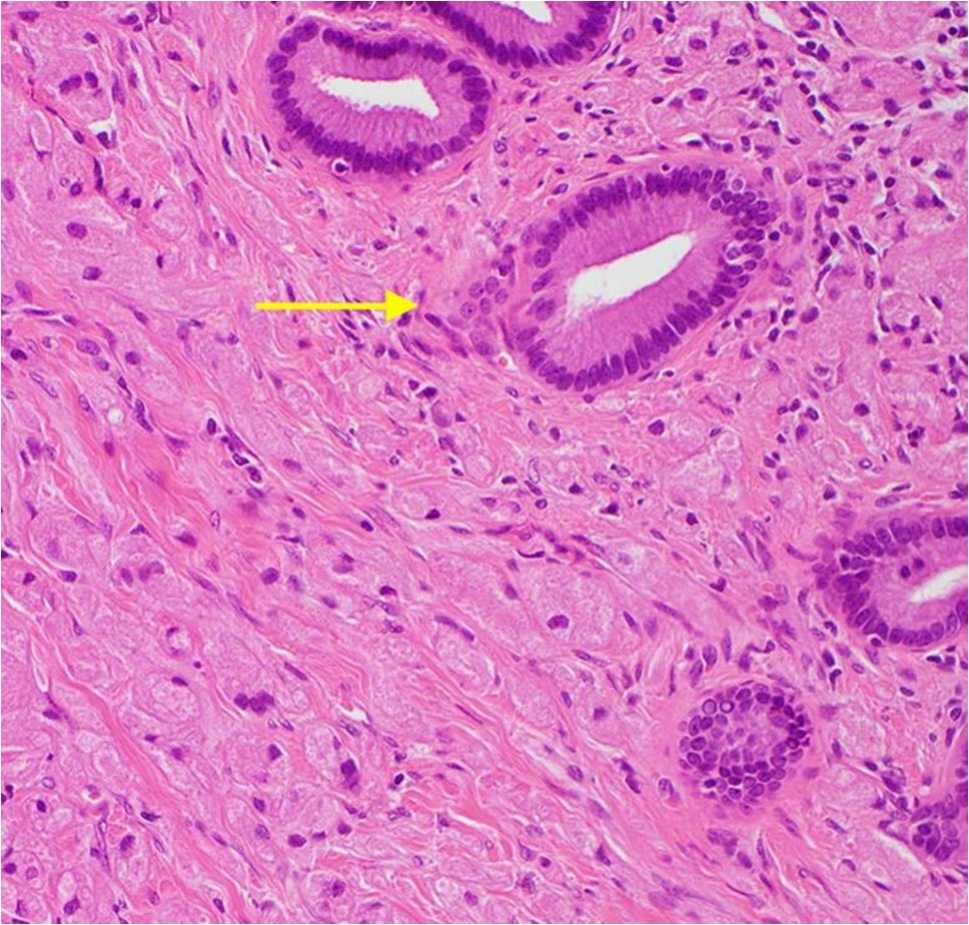
Tumour cells containing abundant granular eosinophilic cytoplasm infiltrating around benign pancreatic ducts (top right) (H&E stain, 200 × magnification)

A 37-year-old male presented with a 1-month history of intermittent, mild upper abdominal pain radiating to the back. An outpatient abdominal ultrasound demonstrated a pancreatic lesion suspected to be an intra-papillary mucinous neoplasm (IPMN), an uncommon diagnosis in this age group. Initial magnetic resonance imaging (MRI) showed no evidence of an enhancing mass though the main pancreatic duct (MPD) was dilated up to 6 mm. There was mild enhancement of the distal pancreatic body and tail in the post-gadolinium images, suggestive of atrophic changes secondary to main duct IPMN. The patient was observed for 6 months and a repeat MRI showed similar findings with unchanged dilatation of the MPD and subjacent pancreatic parenchymal atrophic changes (Fig. 1a and b), but no enhancing mural lesion was seen. There were no features of chronic pancreatitis, no vascular involvement, or metastatic disease, and serum Ca19-9 was normal. Endoscopic ultrasound (EUS) revealed a dilated 6 mm MPD with irregular contours in the main duct from pancreatic body to tail. There was also increased lobularity within the adjacent pancreatic parenchyma and findings were interpreted as suspicious for main duct IPMN. Fine-needle aspiration (FNA) was performed and cytological evaluation showed no malignant cells. Following multidisciplinary team discussion, a spleen-preserving distal pancreatectomy was performed and histopathological examination confirmed complete resection of the tumour. The postoperative course was complicated by an intra-abdominal abscess and a hospital-acquired pneumonia, treated with percutaneous drainage and intravenous antibiotics. The patient was well at 24 months follow up. Histopathological evaluation of the pancreatic resection specimen revealed a 6 mm granular cell tumour (GCT) surrounding the MPD (Figs. 2 and 3). On immunohistochemistry, the tumour cells were diffusely positive for S100. Distal to the tumour, there was marked chronic pancreatitis with dilatation of the MPD and its branches. There was no evidence of cytologic atypia, mitotic activity, or necrosis to suggest malignant transformation as per Fanburg-Smith criteria [8].

## Methods

A systematic review was conducted in accordance with Preferred Reporting Items for Systematic review and Meta-Analysis protocols (PRISMA) statement. Review was registered in PROSPERO ID CRD42022356253. Databases MEDLINE, EMBASE, Scopus, World of Science, PubMed, and the grey literature were searched using MeSH terms with Boolean operators “granular cell tumour” AND “pancreas” up to August 2021. Inclusion criteria included human patients with primary granular cell tumour of the pancreas confirmed on histology. Exclusion criteria were patients without histological diagnosis of GCT defined by S100 positive staining on immunohistochemistry. Full criteria in Table 1.

**Table 1:** Inclusion and exclusion criteria

Inclusion criteria	Exclusion criteria
Human	Guidelines, literature reviews, commentaries, or editorials
All languages	No immunohistochemistry diagnosis of GCT
Case reports, case series, or retrospective reviews	No clinical features reported
Primary GCT of the pancreas	No imaging findings reported
Synchronous disease	

Reference lists and grey literature were examined to broaden search strategy and capture studies. No language, regional, or chronological restrictions were made. Study quality and risk of bias was assessed with the tool by Murad [9] to evaluate methodological quality of case reports and case series. Four domains of patient selection, ascertainment, causality, and reporting were assessed with scoring performed on a 1–5 scale and quality categorised as low (1–2), medium (3–4), and high (5). Three questions in the original questionnaire were removed as irrelevant to this review.

Two authors (SM and KT) assessed studies for inclusion and their quality, with disagreements resolved using an independent third author (KK) by consensus.

Two independent authors (SM and KT) extracted data from studies into Microsoft Excel with outcomes collected including patient background, clinical presentation, investigations, radiological findings, histopathology findings, management, morbidity, mortality, and follow up. Descriptive statistics were used for reported outcomes, with dichotomous variables recorded as percentages and continuous variables as median. Qualitative outcomes for radiology findings were interpreted through narrative synthesis. Statistical analysis of Point-Biserial correlation was performed with IBM SPSS Statistics version 28 [10].

## Results

**Fig. 4:**
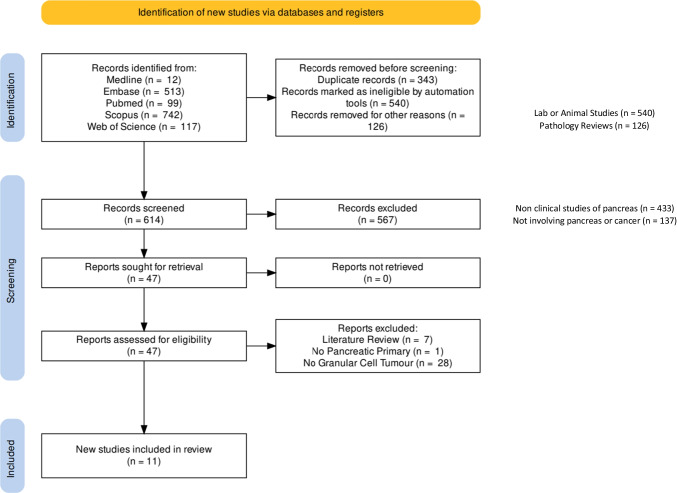
PRISMA flowchart

The search strategy is detailed in the PRISMA flowchart (Fig. 4). 614 studies were identified with 343 duplicates removed. Inclusion and exclusion criteria were applied with 11 relevant case reports were identified for analysis.

Quality of papers assessed demonstrated moderate quality in 5 papers and high in 6 papers (Table 2). Due to the rarity of the disease, all case reports were single patient studies.

**Table 2:** Quality assessment of case reports (1, 11–20)

	Selection	Ascertainment		Causality	Reporting	Total score	Quality
	Do patients represent whole experience of investigator centre?	Was exposure adequately ascertained?	Was outcome adequately ascertained	Was follow-up long enough for outcome to occur?	Is the case described with sufficient detail to allow investigators to make inference to own practise?	1–5	
Wellmann 1975	Yes	Yes	Yes	No	No	3	Moderate
Seidler 1986	Yes	Yes	Yes	Yes	Yes	5	High
Sekas 1988	Yes	Yes	Yes	Yes	Yes	5	High
Nojiri 2001	Yes	Yes	Yes	No	Yes	4	Moderate
Bin-Sagheer 2002	Yes	Yes	Yes	No	No	3	Moderate
Meklati 2005	Yes	Yes	Yes	No	Yes	4	Moderate
Kanno 2010	Yes	Yes	Yes	No	Yes	4	Moderate
Suker 2017	Yes	Yes	Yes	Yes	Yes	5	High
Takahashi 2018	Yes	Yes	Yes	Yes	Yes	5	High
Garves-Descovich 2018	Yes	Yes	Yes	Yes	Yes	5	High
Krutsri 2019	Yes	Yes	Yes	Yes	Yes	5	High

### Demographics

11 case reports were identified with characteristics in Table 3 and summary in Table 4 [1, 3, 11–19]. Diagnosis was confirmed on tissue histopathology based on positive immunohistochemical staining for S-100 protein. Median age was 43 years ranging from 29 to 68 with 36% of patients male. Six patients had no past medical history and only two patients [11, 14] had a history of alcoholic liver disease and chronic pancreatitis. One patient [19] that had a previous GCT diagnosis of the right forearm cured via resection 12 years prior, however, was unable to confirm as metastasis or second pancreatic primary. Median GCT size was 9.5 mm described in ten studies ranging from 6 to 22 mm. Predominant lesion location was pancreatic body (64%) followed by tail and MPD.

**Table 3:** Study characteristics (1, 11–20)

Study	Age and gender	Pancreas location	Size (mm)	Medical history	Other locations	Presenting complaint	EUS findings	CT findings	MRI findings	MPD dilatation	Biopsy	Diagnosis (pre-operative)	Management	Complication	Follow up (months)
Wellmann 1975	29 Male	Head	6	Alcoholic liver disease	Nil	Post mortem (lobar pneumonia)	-	-	-	No	Nil	ND	(Diagnosis after death)	ND	ND
Seidler 1986	62 Female	Tail	7	Bullous pemphigoid, diabetes	Nil	Bullous pemphigoid, diabetes mellitus, abnormal liver function tests	-	Enlarged head of pancreas	-	No	Nil	ND	Distal pancreatectomy	ND	18
Sekas 1988	31 Female	Head	5	Nil	Nil	Abdominal pain and weight loss	-	Prominence of pancreatic head	-	Yes	Nil	ND	Pancreaticojejunostomy	None	20
Nojiri 2001	58 Male	Head	13	Alcoholic hepatitis, chronic pancreatitis	Nil	Back pain, diarrhoea, constipation	-	Pancreatic head hypo-enhancement	-	Yes	Nil	Invasive pancreatic head cancer	Pancreaticoduodenectomy	None	52
Bin-Sagheer 2002	50 Female	Body-tail	-	ND	Stomach	Abdominal pain, weight loss	-	Mass at junction of body and tail of the pancreas	-	Yes	Nil	Pancreatic cancer	Distal pancreatectomy	ND	ND
Meklati 2005	26 Female	Body-tail	5	Nil	Nil	Abdominal pain, weight loss, vomiting	Multiple cystic lesions with necrotic content and thickened walls	Multiple hypo-dense cystic lesions in the tail	Multiple cystic lesions	Yes	EUS FNA	Obstructive pancreatitis secondary to pancreatic duct obstruction of unknown origin	Distal pancreatectomy	None	ND
Kanno 2010	39 Female	Body	22	Nil	Nil	Abdominal pain	Homogenous solid mass with regular hypo-echoic border	Low density lesion with reduced enhancement on early phase and gradual enhancement on delayed phase	Hypo-intense on T1-weighted image. Periphery hypo-intense and central hyper-intense on T2 weighted image	Yes	ERCP cytology	Pancreatic adenocarcinoma	Distal pancreatectomy	None	ND
Sukeri 2017	51 Female	Body	6	Epilepsy, COPD	Yes	Abdominal pain	Dilatation of pancreatic duct and hypo-echoic lesion in pancreas body	Hypo-dense poorly demarcated tumour of the pancreas in the tail-body with impression of infiltrative growth	-	Yes	EUS FNA + brushing cytology	Neuro-endocrine tumour	Distal pancreatectomy	None	12
Takahashi 2018	68 Female	Body	12	ND	Nil	Incidental finding on MRI	Hypo-echoic homogenous pattern with regular borders, poor blood flow, no cystic component	Iso-dense lesion in non-contrast phase, poorly enhanced in arterial, and gradual enhancement from portal vein phase to late phase	Hypo-dense in T1WI, iso-intense in T2WI, hyper-intense in DWI	No	EUS FNA	ND	Non-surgical management	ND	6
Garces-Descovich 2018	43 Male	Body	16	Nil	Caecum	Chest pain, heartburn, increased stooling, haematochezia, weight loss	Well defined sub-centimetre hypo-echoic nodule with dilated MPD	Mild atrophy of distal pancreas with duct dilatation and 1.6 cm slightly hypervascular pancreatic mass in proximal pancreatic body. No local vascular invasion	Segmental dilatation of MPD with irregularity and abrupt non-visualisation at body-tail junction. Parenchymal tail atrophy with loss of intrinsic T1 hyper-intensity. Small pancreatic body enhancing focus on delayed post-gadolinium fat-suppressed T1 weighted images. DWI no restriction	Yes	Colonoscopy	Pancreatic endocrine tumour	Distal pancreatectomy and splenectomy Right hemicolectomy for caecum	None	ND
Krutsri 2019	32 Female	Body	20	DCIS right breast. GCT right forearm (excised). Asymptomatic gallbladder polyps	Nil	Incidental finding on MRI	Hypo-echoic mass with posterior echo enhancement, well-defined border	Hypo-enhancement in the arterial phase, iso-enhancement in the delayed phase. Well-defined border	Hypo-intense in T1W1, hyper-intense on T2W, high signal on DWI	No	EUS FNA	Granular cell tumour (by EUS-FNA) combined with previous GCT	Distal pancreatectomy and splenectomy	None	6
Present study 2021	37 Male	Body	6	Nil	Nil	Abdominal pain	Dilated MPD with irregular contours from body to tail. Increased lobularity within adjacent pancreatic parenchyma	-	Dilatation of MPD with mildly increased enhancement of distal body and tail	Yes	Nil	IPMN	Distal pancreatectomy	Post-operative collection requiring percutaneous drainage under radiological guidance	24

**Table 4:** Summary of study characteristics

	Number/median	%
Age	43	
Male	3	27.3
No past medical history	3	27.3
Abdominal pain	5	45.5
Synchronous disease	2	18.2
Surgical management	10	89.9
Main pancreatic duct dilatation	7	63.6
Granular cell tumour size (mm)	9.5	
Lesion location		
Head	3	27.3
Body	6	54.6
Body and tail	2	18.2
Tail	1	9.1
Follow up (months)	18	

### Symptoms

Seven patients presented with gastrointestinal symptoms, with abdominal pain the main presenting complaint in 45.5% of cases. Two patients were asymptomatic and discovered as incidental findings radiologically [18, 19] although both were being followed up for biliary pathology (pancreatic cyst and gallbladder polyps respectively). One diagnosis was made post-mortem from lobar pneumonia, and one patient [12] presented with a constellation of symptoms and deranged liver function tests leading to investigations for abdominal malignancy.

### Imaging

Except for the 1975 study [11], all patients underwent CT with portal venous contrast and all studies underwent endoscopic examination with cases prior to 2005 undergoing ERCP and after 2010 EUS. Only one patient [3] underwent both EUS and ERCP in order to obtain biopsy via ERCP. Six patients (55%) underwent biopsy; four via EUS, one via ERCP, and one via colonoscopy for synchronous GCT in the ascending colon. Pre-operative diagnosis of pancreatic carcinoma from combined imaging and biopsy results was described in six studies that led to progression to surgical resection. Only one study [18] had a pre-operative histologically confirmed GCT diagnosis on EUS FNA.

Imaging findings (Table 5) were similar across studies for EUS and contrast enhanced CT. EUS demonstrated hypoechoic lesion in five studies with homogenous appearance. On non-contrast CT GCT was iso-enhancing on and with portal venous contrast demonstrated hypo-enhancement that gradually enhanced on late phases. MRI was performed in three studies that demonstrated hypointense lesions on T1W1 and hyperintense on DWI in two studies. T2W demonstrated hyperintensity in two studies; however, one study [18] (9) demonstrated iso-intensity compared to another [3] describing a hypointense periphery only.

**Table 5:** Summary of radiology findings (11–20)

		CT with contrast	MRI T1	MRI T2	MRI DWI	EUS
Nojiri	2001	Hypodense	-			-
Bin-Sagheer	2002		-			
Meklati	2005	Hypodense	-			
Kanno	2010	Hypodense	Hypointense	Central hyperintense		Hypo-echoic
Sukeri	2017	Hypodense				Hypo-echoic
Takahashi	2018	Hypodense Iso-dense on non-contrast phase	Hypointense	Iso intense	Hyper intense	Hypo-echoic
Garces-Descovich	2018	-	Hypointense		Iso intense	Hypo-echoic
Krutsri	2019	Hypodense Iso-dense on delayed phase	Hypointense	Hyperintense	High intense	Hypo-echoic

MPD dilatation was present in 7 patients (64%), of which all 7 patients with gastrointestinal symptoms had MPD dilatation. Point-biserial correlation of GCT size and MPD dilatation showed a negative correlation − 0.07, however, was non-significant (*p* = 0.985). GCT size and abdominal symptoms showed a correlation 0.718 but similarly non-significant (*p* = 0.718).

### Management

10 patients were managed surgically and 1 non-operatively. Two patients had distant disease [1, 15, 20] of which one underwent colonoscopy confirming synchronous caecal disease which was managed with right hemicolectomy, and the other diagnosed via intra-operative biopsies of gastric serosa. No complications were described in 6 studies with median follow up 15 months ranging from 6 to 52 months.

## Discussion

There have only been eleven reported cases of GCT of the pancreas, and with this case report, twelve cases with findings and comparisons included in this systematic review (Table 3).

GCTs are more commonly found in adults, and more than 50% of them are located within body of the pancreas [4, 21]. Although the majority are benign, there have been reports of malignant GCT in 1–2% of cases [22]. Concerning features for malignant GCTs include a large tumour (> 3 cm at diagnosis), rapid growth, and ulceration [23]. The precise histogenesis of these tumours has historically proven challenging; however, there is increasing evidence that GCTs are of neural origin and arise from Schwann cells, given their structural similarities and mutual positivity for the protein S-100 [14]. The S-100 proteins are unique to neural cells and assist in differentiating between tumours of neural and soft-tissue origins [24]. Within the pancreas, GCTs are typically characterised by the presence of abundant granular cytoplasm [25] surrounding the pancreatic duct or its branches (Fig. 3). Pancreatic GCT usually presents as a solitary lesion, is often an incidental finding, usually < 3 cm, and has a favourable prognosis due to the low rate of progression and recurrence [21]. There have been no confirmed reported cases of recurrence once complete excision has been achieved.

### Radiology investigations

The MRI findings of GCT have been previously reported, although without much consistency in its radiological features. In our study, MRI was unable to identify a well-defined mass; however, there was distal main duct dilatation similar to other case reports [3, 12, 13, 20]. As demonstrated cases of pancreatic GCTs exist where main duct dilatation has not been observed; therefore, this finding is not a reliable distinguishing factor for this condition. GCT of the pancreas poses a diagnostic dilemma due to its rarity and no characteristic defining features clinically or radiologically. In the majority of cases, histopathology confirmed GCTs were misdiagnosed preoperatively based on clinical and radiological findings as either suspected pancreatic ductal adenocarcinoma (PDAC) or IPMN. IPMN is often incidentally diagnosed with similar MRI findings to those identified in the present case report, with features of ductal dilatation and the presence of a cystic lesion [26]. This review did identify consistencies in CT and EUS imaging modalities with a hypodense and hypo-echoic lesion described in all cases. Similarly, MRI T1 phase was uniformly hypointense, with inconsistencies in T2 and DWI phasing, however, with no directly opposite contradicting findings. These imaging findings, whilst non-specific, could assist in diagnosis for benign lesions. No malignant pancreatic GCT was identified in this review, and thus no radiological features for rapid growth or high-risk changes could be identified which could be utilised in a surveillance management pathway.

Despite the utility of FNA under EUS guidance in the work-up of pancreatic masses and diagnosis of PDAC, its utility in the diagnosis of pancreatic GCT is unclear. This is arguably due to the limited sample that is obtained from an FNA, preventing from an accurate diagnosis. There have been two cases that have been successfully diagnosed using EUS [18, 19], of which one case study avoided surgical intervention [18]. In all other studies, confirmation of GCT was only made on histopathological assessment of the resected specimen. One study [19] reveals some differentiating features between PDAC and GCT on contrast-enhanced EUS; however, without FNA diagnosis, the definite distinction between the two tumours remains difficult. Improved procedural technique, experience, and technological advancements in the equipment used in EUS-guided FNA may aid in obtaining a more accurate preoperative diagnosis [18, 27]. In doing so, radical dissection may be avoided given the indolent course of this tumour.

### Risk factors

Multiple granular cell tumours have been reported in association with syndromes associated with aberrant RAS/MAPK signalling including Noonan Syndrome and neurofibromatosis [28–31]. However, the great majority of GCT arise sporadically. A clinicopathological study of 110 patients demonstrated a male predominance and ages ranging in the 2nd to 5th decade of life [32]. This review demonstrated similar demographic features with the exception of gender distribution. No risk factor analysis was conducted, as many of the studies examined included limited or non-contributory data on patient medical history. A relationship between tumour size and symptomology or MPD dilatation discerned no significant findings, however, was underpowered due to low patient number.

### Limitations

Limitations of this review owe to the rarity of this disease process within the published literature. Pancreatic GCT is an extremely rare pathology and the diagnostic obscurity of this condition still remains, despite the few case studies that have been reported to date. Subgroup analysis to examine mortality, malignancy, or the natural history could not be performed due to lack of data and short follow up duration. A meta-analysis was planned however not performed as deemed inappropriate due to limited data and clinical and statistical heterogeneity. Study heterogeneity exists owing to the published literature’s broad timespan of 44 years, which is reflected in the distinct change and usage of contemporary imaging modalities EUS and MRI. Quality of review’s case reports was moderate-high; however, the certainty of evidence assessment is low owing to scarce literature limited to case reports.

### Management

Most studies did not elaborate on the decision-making process to proceed with operation, often the presumed reason being suspicion for malignancy. Thus, a comparison could not be performed to non-operative management only performed in one case, which discourages strong recommendations to pursue non-operative management and surveillance. Despite being a predominantly benign tumour with no cases of malignancy reported, a reported 32% risk of recurrence with malignant GCT exists [8]. Without data or reports that detail GCT’s natural history and its potential for malignant transformation, and absence of distinct radiological features that could suggest progression of disease, we suggest surgical resection if not otherwise contra-indicated.

Thus, we recommend initial radiological investigations with MRI and EUS, accompanied by FNA biopsy to confirm diagnosis. In lieu of confirmed diagnosis of GCT, we suggest multidisciplinary discussion prior to proceeding to radical resection due to diagnostic uncertainty encompassing other sinister lesions such as IPMN or malignant GCT.

## Conclusion

In conclusion, the majority of GCTs of the pancreas are only diagnosed on histopathological examination of the resected specimen. GCT of the pancreas should be considered as a diagnosis during the work-up of pancreatic masses and in the presence of pancreatic ductal dilatation. Further improvement in the diagnostic tools and techniques such as EUS may assist in making the correct diagnosis preoperatively and may alter the course of management for the patient. Although surgical resection is currently recommended for GCTs due to malignancy risk, the natural history of this condition in the pancreas is still unclear due to its infrequency. Additional cases would expand the currently available dataset of GCTs of the pancreas.

## Data Availability

The datasets used and/or analysed during the current study are available from the corresponding author on reasonable request.
